# Dynamic Changes in Chemosensory Gene Expression during the *Dendrolimus punctatus* Mating Process

**DOI:** 10.3389/fphys.2017.01127

**Published:** 2018-01-10

**Authors:** Su-fang Zhang, Zhen Zhang, Xiang-bo Kong, Hong-bin Wang, Fu Liu

**Affiliations:** Key Laboratory of Forest Protection, Research Institute of Forest Ecology, Environment and Protection, Chinese Academy of Forestry, State Forestry Administration, Beijing, China

**Keywords:** chemosensory gene, mating, expression dynamic, pheromone receptor, insect olfaction, masson pine moth

## Abstract

The insect chemosensory system is pivotal for interactions with their environments, and moths have especially sensitive olfaction. Exploration of the connection between the plasticity of olfactory-guided and molecular level pathways in insects is important for understanding the olfactory recognition mechanisms of insects. The pine caterpillar moth, *Dendrolimus punctatus* Walker, is a dominant conifer defoliator in China, and mating is the priority for adults of this species, during which sex pheromone recognition and oviposition site location are the main activities; these activities are all closely related to chemosensory genes. Thus, we aimed to identify chemosensory related genes and monitor the spectrum of their dynamic expression during the entire mating process in *D. punctatus*. In this study, we generated transcriptome data from male and female adult *D. punctatus* specimens at four mating stages: eclosion, calling, copulation, and post-coitum. These data were analyzed using bioinformatics tools to identify the major olfactory-related gene families and determine their expression patterns during mating. Levels of odorant binding proteins (OBPs), chemosensory proteins (CSPs), and odorant receptors (ORs) were closely correlated with mating behavior. Comparison with ORs from other *Dendrolimus* and Lepidoptera species led to the discovery of a group of ORs specific to *Dendrolimus*. Furthermore, we identified several genes encoding OBPs and ORs that were upregulated after mating in females; these genes may mediate the location of host plants for oviposition via plant-emitted volatiles. This work will facilitate functional research into *D. punctatus* chemosensory genes, provide information about the relationship between chemosensory genes and important physiological activities, and promote research into the mechanisms underlying insect olfactory recognition.

## Introduction

Masson pine (*Pinus massoniana* L.) is a dominant and native forest plant species in southern China. As it grows readily in poor soils, huge forests of this species were planted in southern China; however, these vast areas of *P. massoniana* monoculture forest present problems, including frequent damage by forest insects. One of the most serious pests of coniferous forests in southern China is the pine caterpillar moth, *Dendrolimus punctatus* Walker (Lepidoptera: Lasiocampidae) (Xiao, 1992). During outbreaks, high population densities of *D. punctatus* larvae feed intensively on pine needles, causing substantial damage to trees, and huge economic losses (Zhao et al., 1993). In the past, chemical insecticides were used to treat outbreaks, causing severe negative effects on the biodiversity of the ecosystem (Kong et al., 2007). Thus, the control of the pine caterpillar moth has been of long-term interest to forest insect researchers in China, and new methods are imperative to control this pest. Based on its potential in population outbreak monitoring and pest controlling of olfactory communication system, it attracted the interesting of many scientists (Gao et al., 2001; Kong et al., 2006; Li et al., 2015); however, only fragmentary data is available regarding the molecular mechanisms of odor detection in *Dendrolimus* species (Zhang S.-F. et al., 2014).

Lepidopteran species have highly specific and sensitive olfactory systems (Zhang et al., 2015). Several groups of olfactory-related genes play critical roles in the transformation of chemical signals (such as sex pheromones or plant volatiles) to electrical nervous impulses, including three receptor families, two binding protein families, and the sensory neuron membrane proteins (SNMPs) (Vogt et al., 2009; Zhang et al., 2014a). The three receptor families, odorant receptors (OR), ionotropic receptors (IR), and gustatory receptors (GR), are transmembrane molecules expressed in the sensillar neurons of insect antennae (Kwon et al., 2007; Benton et al., 2009; Robertson and Kent, 2009; Touhara and Vosshall, 2009; Kaupp, 2010). The two binding protein families include odorant binding proteins (OBPs) and chemosensory proteins (CSPs), which are small soluble proteins expressed in the lymph of antennae (Vogt, 2003; Pelosi et al., 2006; Sanchez-Gracia et al., 2009). These two classes of protein also have other functions, as recently reviewed by Pelosi et al. (2017). Classic OBPs contain six conserved cysteine residues, and there are two other type of OBPs, plus-C OBPs, which contain 4–6 additional cysteines, and minus-C OBPs, which contain fewer cysteine residues (generally C2 and C5 are absent) (Hekmat-Scafe et al., 2002; Sanchez-Gracia et al., 2009). Most of the genes encoding these proteins exhibit considerable sequence diversity (Krieger et al., 2004; Robertson and Wanner, 2006; Engsontia et al., 2008; Tanaka et al., 2009), and their identification has primarily been based on genomic data (Zhou et al., 2006, 2008; Gong et al., 2009), or antennal transcriptomes (Grosse-Wilde et al., 2011; Legeai et al., 2011; Bengtsson et al., 2012; Khan et al., 2013; Zhang et al., 2014a; Zhou et al., 2015).

Many moths exhibit olfactory-guided behavioral plasticity, depending on the physiological status of the individual (Anton et al., 2007). In particular, mating can dramatically influence the olfactory behavior of moths. For example, virgin *Vitacea polistiformis* males exhibited four-fold higher electroantennogram responses to pheromones than mated males (Pearson and Schal, 1999). Moreover, only mated *Amyelois transitella* (Walker) (Phelan and Baker, 1987), *Lobesia botrana* (Masante-Roca et al., 2007), and *Manduca sexta* (Mechaber et al., 2002) females, but not virgins, were attracted by plant volatiles. The response of mated *Plutella xylostella* females to some green leaf volatiles was stronger than those of males or unmated females (Reddy and Guerrero, 2000). Clearly, mating can influence the behavioral responses of insects to volatiles, although to differing extents among species. Moths can also adjust the level of chemosensory gene expression depending on their physiological status or development stage. For example, changes in the expression levels of pheromone binding protein 1 correlated with the mating status of *P. xylostella* (Zhang et al., 2009). Another study showed that mating did not affect the expression of minus-C OBPs in male *Batocera horsfieldi* beetles; however, it could affect that of females. Nevertheless, to date, studies attempting to correlate physiological status with dynamic olfactory gene expression remain rare, and this topic warrants further attention.

The majority of *D. punctatus* insects of both sexes only mate once in their lives, while a few mate twice, and mating lasts ~18 h (Zhou, 2013). Furthermore, *D. punctatus* adults do not eat and die soon after oviposition. Thus, mating is the priority for adult *D. punctatus*, and sex pheromone recognition and oviposition site location are their main activities. Notably, these activities are both closely related to olfaction. Thus, the dynamics of chemosensory gene expression during the mating process deserves further study. In general, the numbers of chemosensory genes (such as OBPs and ORs) are huge in insects; for example, there are 44 OBPs and 72 ORs in *Bombyx mori* (Khan et al., 2013), and the specific function of each gene remains unclear. The expression patterns of these genes provide important clues about their functions, and olfactory genes with expression levels closely related to mating and oviposition activities may perform important functions during these behaviors. In this study, we focused on two aims: first, based on our previous work (Zhang et al., 2017), identification of chemosensory genes in *D. punctatus*; second, monitoring the dynamic expression spectrum of chemosensory genes during the whole mating process, with the aim of inferring the functions of different genes. This work will not only facilitate follow-up functional investigation of chemosensory genes, which has potential to identify novel targets for pest control, but also determine the relationship between the spectrum of chemosensory genes and important physiological and behavioral activities, and promote research into the mechanisms underlying insect olfactory recognition.

## Materials and methods

### Insects

In 2015, we collected about 200 *D. punctatus* pupae in Quanzhou, Guilin City, Guangxi province, China, and reared them in our laboratory at 26 ± 2°C, 50 ± 10% relative humidity, and a 16 h light: 8 h dark photoperiod. Male and female insects representing four different physiological conditions were prepared for transcriptome sequencing as follows: newly emerged (within 5 h after emergence, unmated; eclosion), calling females and corresponding males, mating status (copulating), and after mating status (post-coitum). Male and female insects were kept in two different insect rearing cages placed in close proximity to each other and separated by only two layers of screen cloth, so that males could sense the female sex pheromones. Antennae from 15 female and male *D. punctatus* specimens at each stage were cut off and immediately frozen in liquid nitrogen. Insect antennae from each group were divided into three equal parts, as three biological replicates. Thus, in total, we constructed 24 libraries for RNA-seq (four conditions for male and female insects respectively, and three replications for each status).

### RNA-seq library preparation

As previously described (Zhang et al., 2014a, 2017), total RNA samples were extracted using TRIzol reagent (Invitrogen, Carlsbad, CA, USA) and treated with RNase-free DNase I (TaKaRa, Dalian, Liaoning, China). Subsequently, RNA purity, concentration, and integrity were checked using the NanoPhotometer® spectrophotometer (IMPLEN, CA, USA), a Qubit® RNA Assay Kit and a Qubit® 2.0 Flurometer (Life Technologies, CA, USA), and the RNA Nano 6000 Assay Kit on the Bioanalyzer 2100 system (Agilent Technologies, CA, USA), respectively.

Duplex-specific-nuclease normalized cDNA was synthetized using 3 μg total RNA samples (Zhulidov et al., 2004; Bogdanova et al., 2008). The RIN values of all samples were > 8. We prepared sequencing libraries using an Illumina TruSeq™ RNA Sample Preparation Kit (Illumina, San Diego, CA, USA), and added four index codes to identify sequences from each sample. To preferentially select cDNA fragments of 200 bp length, we purified the libraries using the AMPure XP system (Beckman Coulter, Beverly, MA, USA). PCR (10 cycles) was performed to enrich for the two-end adaptor ligated DNA fragments. Finally, the products were purified using an AMPure XP system and quantified on an Agilent Bioanalyzer 2100.

### Clustering and sequencing

Index-coded samples were clustered using the TruSeq PE Cluster Kit v3-cBot-HS (Illumina) on a cBot Cluster Generation System, then sequencing performed on an Illumina Hiseq 2500 platform, according to the manufacturer's instructions.

### *De Novo* assembly

Raw sequencing data were filtered to remove reads containing adapter sequence, reads with > 10% N (uncertain bases), and sequences with error rates > 1% for more than 50%, using self-written Perl scripts, to obtain clean data. Then we calculated the Q20, Q30, GC-content, and sequence duplication level of the clean data, which were subsequently used for downstream analyses. Clean data sequences were compared with the NT database to determine whether they were polluted. Trinity (vesion:trinityrnaseq_r20131110) was used to perform transcriptome assembly (Grabherr et al., 2011). TGICL software was used to reduce redundancy (Pertea et al., 2003). The raw data from our experiments have been deposited in the NCBI SRA database under the accession number SRP102206 (Bioproject accession number PRJNA374901). We assessed the transcriptome assembly using benchmarking universal single-copy orthologs (BUSCO) based on the percentage of sequences aligned with highly conserved protein sequences, (Simão et al., 2015).

### Annotation

First, transcript sequences were searched using BLAST against the NR, SWISSPROT, KEGG, and KOG databases, with a cut-off value of 1e-5, and the highest sequence similarity targets selected for functional annotation of the transcripts. Next, Blast2GO was used to perform GO annotation of the transcripts (Conesa et al., 2005; Götz et al., 2008). Finally, the molecular function, biological process, and cellular component of the genes were assigned (Ashburner et al., 2000; Krieger et al., 2004).

Based on our previous research (Zhang et al., 2017), we further identified the chemosensory genes in *D. punctatus*. Previously identified chemosensory genes were confirmed in our new transcriptome database using tBLASTx searches, and the complete sequences of some previously identified partial genes obtained. We further identified some new chemosensory genes by contig tBLASTx searches. The open reading frames (ORFs) of possible genes were verified by additional BLAST searches (http://blast.ncbi.nlm.nih.gov/Blast.cgi). Newly identified olfactory genes were submitted to GenBank; the updated accession numbers are listed in Table S1. Maximum likelihood (ML) and neighbor-joining (NJ) phylogenetic trees of chemosensory genes were constructed using MEGA5 with 1,000 bootstrap replications (Tamura et al., 2011). MEGA's model test was used to select the best model for ML tree construction. Dendrograms were colored in Adobe illustrator (Adobe Systems). Motif analysis of the predicted intact ORFs of chemosensory genes was performed using the MEME online server (Version 4.12.0.) (Bailey et al., 2009) http://meme-suite.org/tools/meme. For OBP and CSP, the motif discovery parameters were: minimum width = 6, maximum = 10, maximum motifs to find = 8; for ORs they were: minimum width = 15, maximum = 50, maximum motifs to find = 8.

### Gene expression quantification

To measure the gene expression levels in transcriptomes, we used the FPKM (fragments per kilobase of exon per million fragments mapped) criteria (Trapnell et al., 2010). Three biological replicates were sequenced for each *D. punctatus* status, and the mean FPKM value and standard error obtained from the three replicates. Differentially expressed genes (DEGs) between different mating status insects were calculated using DESeq (http://bioconductor.org/packages/release/bioc/html/DESeq.html) (Anders and Huber, 2010), based on the reads of each unigene. Unigene expression levels and DEGs were normalized following the compatible-hits-norm model (Bullard et al., 2010). DEGs were screened to identify those generating *q*-values ≤ 0.05 using the false discovery rate (FDR) method (Noble, 2009).

### GO enrichment and expression trend analysis of DEGs

GO Enrichment analysis of DEGs was carried out using GOstat (Beißbarth and Speed, 2004), with *p*-values approximated using Chi-square tests, with all annotated genes used as the background. Short Time-series Expression Miner (STEM, vision 1.3.11) was used to analyze the expression trends of some DEGs (Ernst and Bar-Joseph, 2006). FPKM values were log2 transformed and imported into the software.

### Quantitative real-time PCR (qPCR)

qPCR was carried out to validate the RNA-Seq data, similar to our previous report (Zhang et al., 2014a,b, 2017). qPCR primers (Table S2) were designed based on cDNA sequences. RT-PCR was performed to test whether qPCR primers could amplify the correct products. Beta-actin was used as the housekeeping gene. T-easy clones containing the tested genes were constructed as reference genes to construct qPCR standard curves. Amplification efficiencies of all primers tested were 90–100%. Real-time PCR was carried out in a Roche LightCycler 480 (Stratagene, La Jolla, CA, USA). The PCR cycles were as follows: 2 min at 95°C, 40 cycles of 20 s at 95°C, 20 s at 58°C, and 20 s at 72°C; finally, melting curve analysis (58 to 95°C) was performed to evaluate the specificity of the PCR products. Ct values were calculated using the Roche qPCR software (version 1.5.1) with the second derivative method. Three independent biological reactions were completed for each insect status, along with three technical replicates for each reaction. Gene expression levels tested by qPCR in female and male *D. punctatus* antennae (relative to that of the actin gene) were compared with the transcriptome expression data (FPKM), as illustrated in Figure S1.

## Results

### Assembly

Transcriptomic sequence data were generated from antenna cDNA libraries from *D. punctatus* adults at different mating stages using Illumina HiSeq™2500 technology. We acquired 204.30 Gbp of clean sequence data in 1,634,361,960 clean reads. After assembly, 110,760 unigenes were obtained, with an N50 of 2,380 bp. Approximately 80% of unigenes were >500 bp, with a maximum length of 54,680 bp (Figure S2). We evaluated the completeness and accuracy of transcriptome assembly using BUSCOs, and the results demonstrated 98.1% complete BUSCOs (C: 98.1 [S: 83.8%, D: 14.3%], F: 1.0%, M: 0.9%).

### Annotation, GO enrichment, and STEM analyses

**Figure 1:**
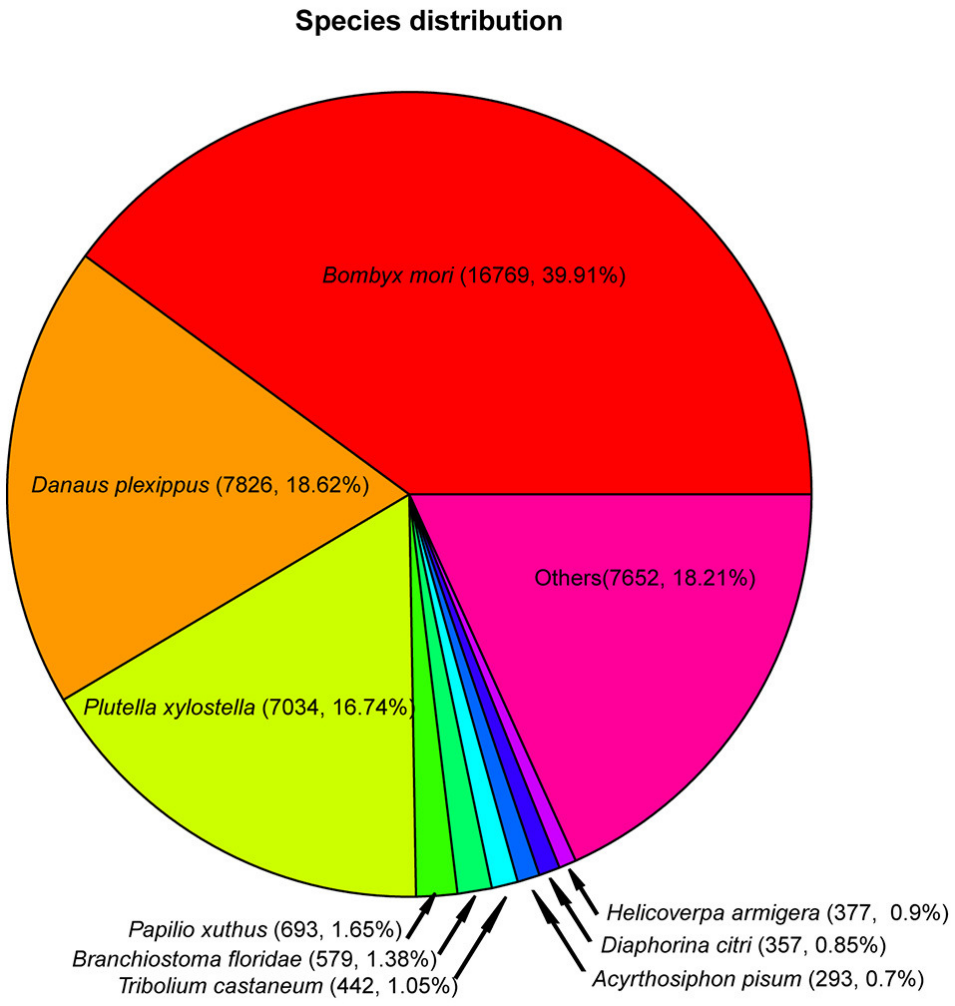
Targets species distribution of annotated *D. punctatus* contigs, determined using BLAST.

BLAST analysis indicated that *D. punctatus* transcriptome unigenes were most similar to amino acid sequences from three other Lepidoptera species: *B. mori* (16,769 hits with E-values <1e-5), *Danaus plexippus* (7826 hits with E-values <1e-5), and *P. xylostella* (7034 hits with E-values <1e-5) (Figure 1). These three species accounted for ~75% of hits.

Function distribution, determined by Gene Ontology (GO) analysis, indicated that *D. punctatus* genes were primarily enriched for binding or catalytic activity (Figure S3A), and followed by transporter and structural molecule activity. KOG classification indicated that genes involved in signal transduction occupied an important position (Figure S3B).

**Figure 2:**
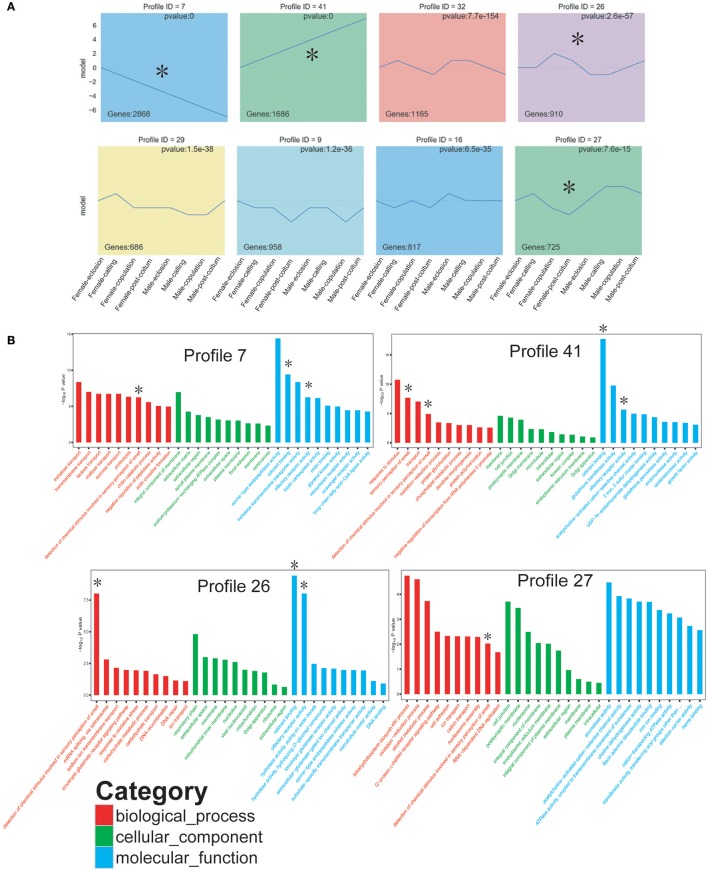
Short Time-series Expression Miner (STEM) analyses of differentially expressed unigenes (DEGs). **(A)** Significant profiles. **(B)** GO enrichment of profiles. Items marked with asterisks are associated with insect chemo-sensation.

GO enrichment analysis of DEGs revealed that waves of gene expression changes occurred in the antennae of *D. punctatus* during the mating process (Figure S4). Overall, olfactory detection genes, particularly olfactory receptors and odorant binding related genes, exhibited dramatic differences between sexes and also during the mating process of male and female insects. To further elucidate the characteristics of gene expression in insects of different mating status, we analyzed the expression trend of DEGs with short time-series expression miner (STEM). Thirteen significant profiles were obtained, four of which were related to chemosensory genes (Figure 2A). GO enrichment of the profiles indicated that profiles 41 and 26 included the most chemosensory associated genes (Figure 2B). Of the four chemosensory related profiles, profile 7 continually declined and included genes expressed more highly in female antennae; profile 41 continually rose, and included genes expressed at higher levels in male antennae; profiles 26 and 27 fluctuated in the eight *D. punctatus* groups, and included genes with expression levels that oscillated during the mating process of this insect.

### Identification and expression dynamics of chemosensory genes

GO enrichment and STEM analysis indicated that chemosensory genes may be very important in the mating process. Thus, detailed analyses were performed to determine the characteristics of the olfactory-related gene families identified from the transcriptomes of *D. punctatus* in different mating states.

In our previous work, we identified a considerable number of *D. punctatus* chemosensory genes (Zhang et al., 2017). Here, after further effort, the complete sequences of many of the partial gene sequences identified previously were acquired, including six OBPs (NCBI accession numbers KY225481–KY225486), one CSP (KY225487), 23 ORs (KY225488–KY225510), one GR (KY225519), and eight IRs (KY225529–KY225536). Some new genes were also identified, including eight ORs (KY225511–KY225518), nine GRs (KY225520–KY225528), and five IRs (KY225537–KY225541).

**Figure 3:**
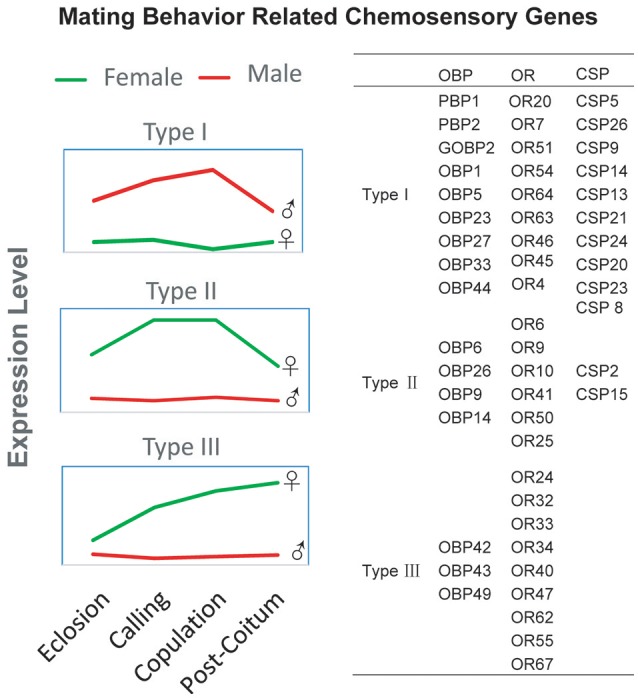
Expression model of sex-biased chemosensory genes in *D. punctatus* with different mating status. **(Left)** Three expression model types of sex-biased gene expression in different mating states. **(Right)**, OBP, OR, and CSP genes belonging to each of the three models.

The correlation between the expression levels of chemosensory genes and mating status was examined in detail, and the expression levels of OBPs (Figure S5), CSPs (Figure S6), ORs (Figure S7), GRs (Figure S8), and IRs (Figure S9) determined. Further analysis indicated that the chemosensory genes exhibited different expression levels in insects in different mating states, with three different patterns identified (Figure 3). First (Type I), some genes were more strongly expressed in male than female antennae. In general, these genes were upregulated during calling or mating, and downregulated after mating. There were nine OBPs (Figure 3, Figure S5A); ten CSPs (Figure S6A); and nine ORs (Figure S7A) in this category. Second (Type II) were genes expressed at higher levels in female than male antennae. These genes were generally upregulated during calling or mating, and downregulated after mating (Figure 3). This category consisted of four OBPs (indicated as red in Figure S5B), two CSPs (Figure S6B), and six ORs (Figure S7B). Third (Type III) were genes expressed at higher levels in female than male antennae, and continually upregulated over time (Figure S3). This third category contained three OBPs (green in Figure S5B) and nine ORs (green in Figure S7B). To further confirm the expression level of olfactory genes, the ORs (Figure S1A) and OBPs (Figure S1B) that were belong to type I were selected for qPCR verification of the transcriptome expression data, and the results indicated that the transcriptome expression data were credible (Figure S1).

**Figure 4:**
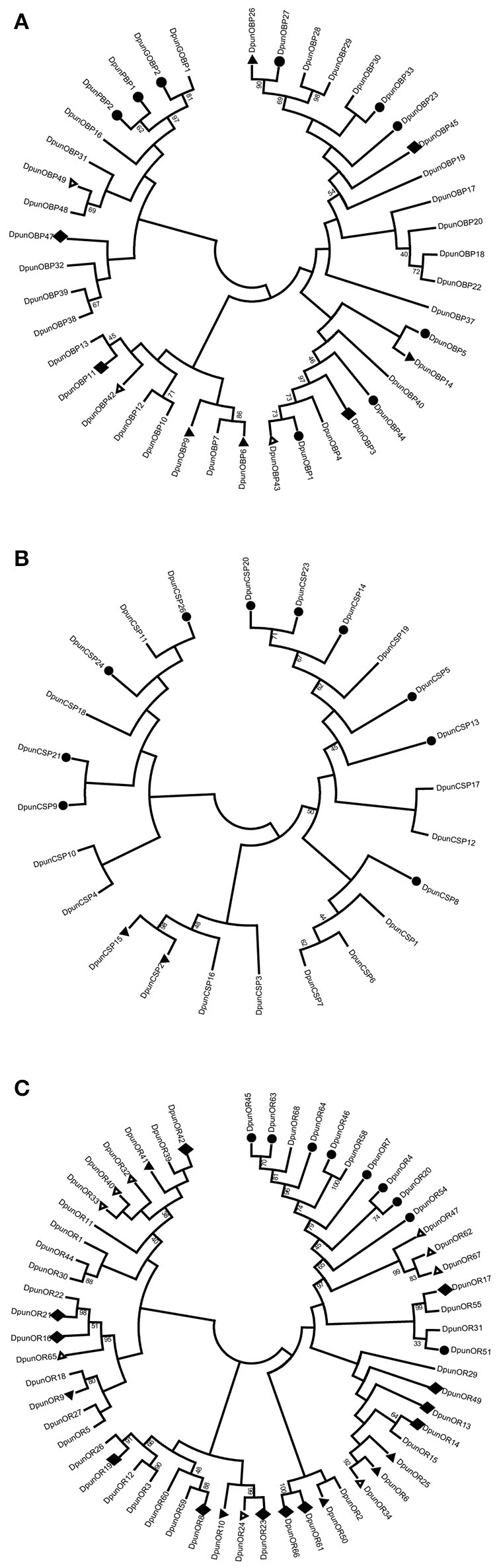
Phylogenetic analysis of the chemosensory genes in *D. punctatus*. Neighbor-Joining dendrograms based on protein sequences of candidate odorant-binding proteins (OBPs) **(A)**, candidate chemosensory proteins (CSPs) **(B)**, and odorant receptors (ORs) **(C)**. Bootstrap consensus trees were inferred from 1,000 replicates. Branches corresponding to partitions reproduced in less than 40% bootstrap replicates were collapsed. Proteins expressed at higher levels in male antennae are indicated by solid circles; those expressed at higher levels in female antennae are indicated by filled triangles (higher expression during calling or mating, and downregulated after mating), hollow triangles (continually upregulated over time in female antennae), and squares (other genes expressed at higher levels in female than male antennae).

Next, phylogenetic analyses of chemosensory genes identified from *D. punctatus* were performed. A phylogenetic tree of the identified OBPs was constructed (Figure 4A). Unsurprisingly, PBP1, PBP2, and two GOBPs were grouped together, and three of these four genes were expressed at higher levels in male than female antennae. Interestingly, other OBPs more strongly expressed in male antennae were all grouped with an OBP that was preferentially expressed in female antennae. Phylogenetic analysis indicated that CSPs expressed at higher levels in male antennae (indicated by solid circles) were dispersed into two subclasses in the tree, while CSPs expressed more strongly in female antennae (filled triangles) were grouped separately (Figure 4B). Interestingly, the male biased ORs were almost all clustered in a single branch (Figure 4C).

**Figure 5:**
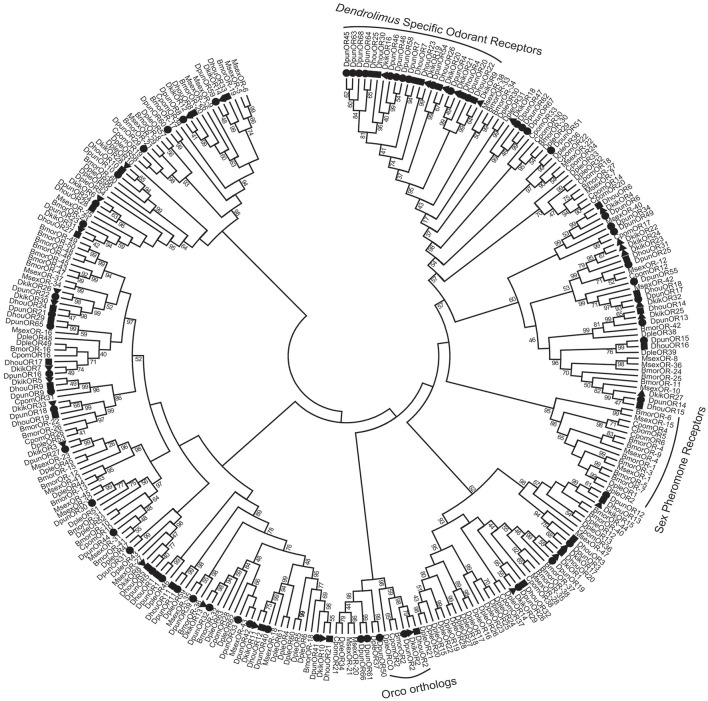
Maximum likelihood dendrogram based on protein sequences of candidate odorant receptors (ORs) in *D. punctatus* and other insects. Evolutionary history was inferred using the Maximum likelihood method. The bootstrap consensus tree inferred from 500 replicates was taken to represent the evolutionary history of the taxa analyzed. Branches corresponding to partitions reproduced in less than 40% of bootstrap replicates were collapsed. ORs from *D. punctatus* (Dpun), *Dendrolimus houi* (Dhou), *Dendrolimus kikuchii* (Dkik), *Bombyx mori* (Bmor), *Manduca sexta* (Msex), *Danaus plexippus* (Dple), and *Cydia pomonella* (Cpom) were included. The Orco orthologs, pheromone receptor subfamily genes, and *Dendrolimus* specific odorant receptors are indicated on the figure.

To further analyze the characteristics of *D. punctatus* ORs, we performed phylogenetic analysis including ORs from two sister species of *D. punctatus, Dendrolimus houi*, and *Dendrolimus kikuchii* (Zhang et al., 2014a), and four other Lepidopteran species, including *B. mori, D. plexippus, M. sexta* (Grosse-Wilde et al., 2011), and *Cydia pomonella* (Bengtsson et al., 2012). The results permitted several observations (Figure 5): first, the co-receptor Orco was identified in *D. punctatus* and was conserved among these moths; second, the ORs from *D. punctatus* generally formed small subgroups together with those of *D. houi* and *D. kikuchii*, and sometimes with *B. mori* and *M. sexta*; third, the sex pheromone receptors from *B. mori, M. sexta, D. plexippus*, and *C. pomonella* formed clade in the tree (labeled “sex pheromone receptors” in Figure 5); however, none of the ORs from the three *Dendrolimus* species were clustered in this group; finally, a group of ORs from *D. punctatus, D. houi*, and *D. kikuchii* (labeled “*Dendrolimus* Specific Odorant Receptors” in Figure 5) formed a subgroup that included no receptors from the other moths, this is unusual in the Lepidoptera, and the specific functions of these ORs require further investigation.

### Motif-pattern analysis of OBPs, CSPs, and ORs

**Figure 6:**
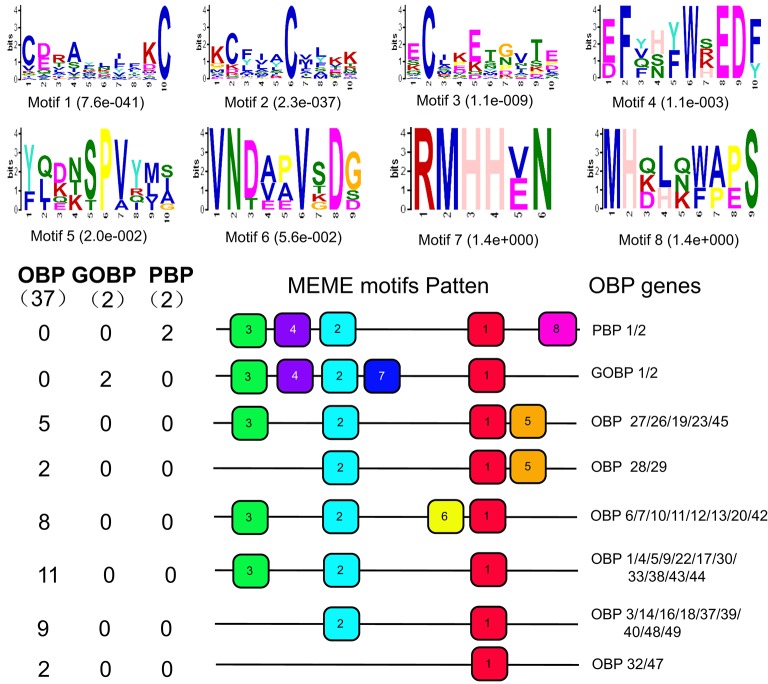
Motif analysis of OBP genes from *D. punctatus* with different mating status. **(Above)** The eight motifs that were most frequently identified in the investigated protein sequences. **(Below)** The approximate location of each motif in the protein sequences. The numbers in the boxes correspond to the numbered motifs in the upper part of the figure.

To further understand the sequence characteristics of the chemosensory genes in *D. punctatus*, we performed motif-pattern analysis of OBPs, CSPs, and ORs using the MEME server. OBP motif analysis revealed eight groups (Figure 6). Motif 1 was contained in all OBPs. All GOBPs and PBPs shared four motifs 1–4, while the eighth motif was exclusive to PBPs, and the seventh motif was only found in GOBPs. OBPs in the third and fourth groups that contain the fifth motif were all minus-C OBPs, indicating that Motif 5 is a characteristic of minus-C OBPs. The eighth group included two plus-C OBPs.

**Figure 7:**
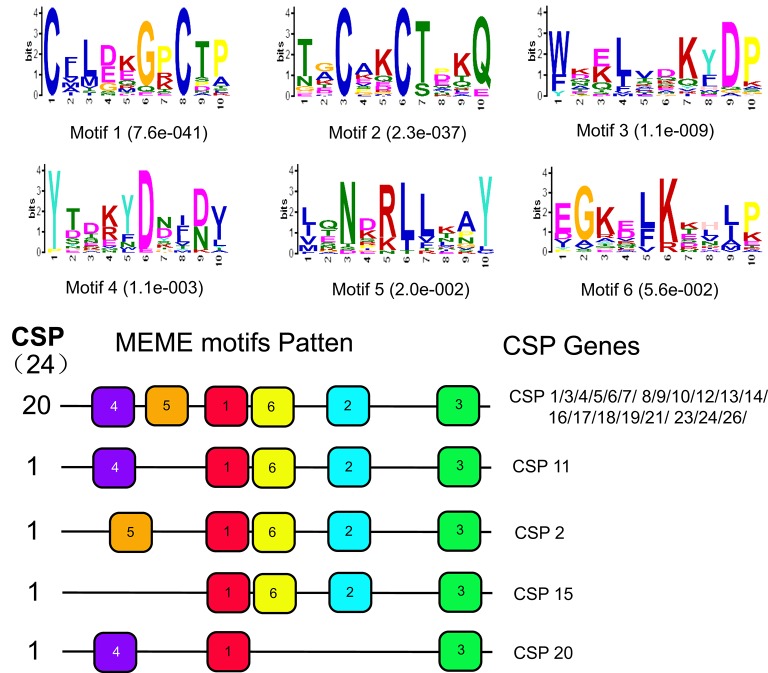
Motif analysis of CSP genes from *D. punctatus* with different mating status. **(Above)** Six motifs most frequently identified in the investigated proteins. **(Below)** The approximate location of each motif in the protein sequences. Numbers in boxes correspond to the numbered motifs in the upper part of the figure.

Motif analysis of CSP sequences indicated that they were relatively conserved, with the majority containing the same motif pattern, with some exceptions (Figure 7). Interestingly, two CSPs (CSP2 and CSP15) that contained different motif patterns with respect to the others were those expressed at higher levels in female antennae.

**Figure 8:**
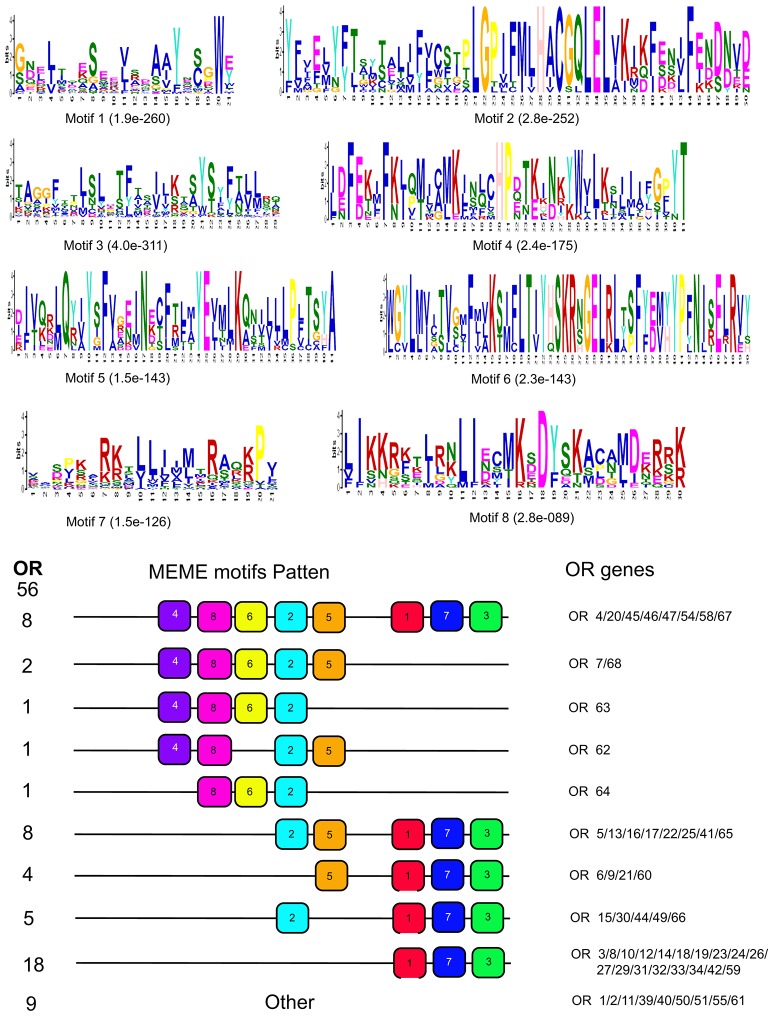
Motif analysis of OR genes from *D. punctatus* with different mating status. **(Above)** Eight motifs that were identified most frequently in the investigated proteins. **(Below)** The approximate location of each motif in the protein sequences. The numbers in the boxes correspond to the numbered motifs in the upper part of the figure.

OR motif analysis indicated that the majority of sequences could be separated into nine groups (Figure 8), depending on their motif patterns. We designated the first five groups as class 1, and the sixth to ninth groups as class 2, as the conserved motifs of class 1 were concentrated at the 5′ end of the genes, while those of class 2 were at the 3′ end. Comparative analyses indicated that male biased ORs (Figure 3, Type I) all belong to class 1, except for OR51, while female bias ORs (Figure 3, Types II and III) all belong to class 2, other than OR62. Overall, the motif patterns and expression biases of these genes indicated their functional differentiation.

## Discussion

Deciphering the functions of the multiple olfactory-related genes of insects is critical to understanding the olfactory recognition mechanisms of these animals. As important activities of adult insects, mating behaviors rely heavily on sensory systems (Ziegler et al., 2013; Zhang et al., 2015). The chemosensory genes involved in these processes represent a logical starting point for functional analysis of these numerous chemosensory genes. Here, we provide a relatively comprehensive account of the dynamic expression spectrum of the chemosensory genes of *D. punctatus* antennal transcriptomes from different mating conditions. We analyzed the expression patterns of different olfactory genes during the mating process, and discuss the relationship between different genes and mating behaviors. The results have the potential to improve our understanding of the correlations between olfactory gene expression and mating behavior.

The numbers of chemosensory genes identified in this study were much higher than those previously identified in *D. houi* and *D. kikuchii* (Zhang et al., 2014a). For example, we identified 42 OBPs and 58 ORs in *D. punctatus*, while the numbers were 23 and 33 in *D. houi* and 27 and 33 in *D. kikuchii*, respectively. The reason for this discrepancy may be that we constructed more than one transcriptome from *D. punctatus* antennae in different mating states, resulting in greatly improved detection of olfactory genes. Possibly for the same reason, we also identified some new genes with respect to our previous work, which also focused on *D. punctatus* (Zhang et al., 2017). Comparisons with the olfactory gene numbers in other Lepidoptera species, including *B. mori* (44 OBPs, 72 ORs), *M. sexta* (47 ORs), *Spodoptera littoralis* (47 ORs), and *Sesamia inferens* (39 ORs) (Khan et al., 2013), indicate that we obtained a relatively intact chemosensory gene pool for *D. punctatus*. Surprisingly, we only identified two PBPs in *D. punctatus*, consistent with reports for *D. houi* and *D. kikuchii* (Zhang et al., 2014a), which is unlike many other Lepidoptera species which typically have three PBPs (Maida et al., 2000; Abraham et al., 2005; Legeai et al., 2011; Guo et al., 2012; Khan et al., 2013). Simultaneously, no male-specific pheromone receptors were detected through phylogenetic analysis, similar to *D. houi* and *D. kikuchii* (Zhang et al., 2014a). This provides further evidence that the pheromone recognition genes of *Dendrolimus* exhibit characteristic features. Further studies, including PBP and OR ligand binding tests, are urgently required to explore the pheromone recognition mechanisms of *Dendrolimus*.

The identified expression patterns of olfactory genes during the mating process are interesting, and several genes showed different patterns of expression. Several of these olfactory genes were expressed at higher levels in male than female antennae, and were generally upregulated when calling or mating, and downregulated after mating (Type I). The expression levels of genes in this category appear to be correlated closely with mating activity, and it includes a considerable number of OBP, CSP, and OR genes belong to this category. We deduced that these genes can bind or recognize pheromones, or other odors, that are crucial during mating behaviors, leading to physiological responses of insects during mating. Similar results have been reported for *Anopheles gambiae* females, in which one odorant receptor is downregulated after insects have taken a blood meal (Fox et al., 2001). Moreover, behavioral and physiological influences on gene expression levels have been identified in *Drosophila melanogaster* and *Caenorhabditis elegans* (Peckol et al., 2001; Zhou et al., 2009). Interestingly, on phylogenetic analysis, the ORs belonging to this category in *D. punctatus* were almost all clustered into a single branch (Figure 4C); this *D. punctatus* male-antenna biased OR branch in Figure 4C corresponded to the ORs marked “*Dendrolimus* Specific Odorant Receptors” in Figure 5. Although these genes were not clustered into pheromone receptor branches (Figure 5), we strongly suspect that these ORs may be responsible for recognition of *D. punctatus* sex pheromones, although further functional experiments are needed to confirm this hypothesis. Furthermore, we deduced that the sex pheromone receptors of *Dendrolimus* were characteristic of this genus and different from those of other moths.

The genes expressed at higher levels in female than male antennae were also important categories (Type II and Type III). Interestingly, nine ORs and three OBPs (Figure 3) were continually upregulated over time, with peak expression after mating (Type III). We deduced that these genes were correlated with activity after mating, which for *D. punctatus* is oviposition, since adults of this species do not eat or drink, and mating and oviposition are the two primary behaviors of females. During the process of insect oviposition, finding a suitable location is highly dependent on olfaction (de Bruyne and Baker, 2008; Afify and Galizia, 2015). Thus, genes in this category may recognize host plant volatiles, enabling insects to identify suitable locations for oviposition. For example, behavioral evidence from other insects indicate that mated *P. xylostella* females respond more sensitively to green leaf volatiles (Reddy and Guerrero, 2000) and that mated *A. transitella* and *M. sexta* females were attracted by plant volatiles (Phelan and Baker, 1987; Mechaber et al., 2002). Thus, our future functional gene investigations of the ligands of these olfactory genes may focus on host plant volatiles.

Another gene expression pattern, those highly expressed in female antennae during calling or mating, and downregulated after mating (Type II) attracted our interest. Some studies have demonstrated that male moths, such as *Anticarsia gemmatalis*, can also release pheromones (Heath et al., 1983, 1988). To date, the recognition mechanisms of these male released pheromones by female moths are unclear. Since the expression pattern of Type II olfactory genes was observed to be closely correlated with mating activities, these molecules may be important for recognition of pheromones released from males; however, male pheromones released by *D. punctatus* have yet to be identified, hence the function of Type II olfactory genes requires further investigation.

The expression dynamics of CSPs, GRs, and IRs during mating behavior was complex. Several CSPs were correlated with the mating process, including CSP5, 14, 26. Mating related functions of CSPs have been identified by other studies (Zhang Y. N. et al., 2014); however, it seems the identified GRs and IRs only fluctuate mildly during the mating process. These results may coincide with the functions of the encoded proteins. For example, in *Drosophila*, antennal IRs mainly respond to acids, aromatics, and nitrogen-containing compounds (Abuin et al., 2011), while GRs primarily respond to sugars, detergents, salts, and CO_2_, among other substances (Agnihotri et al., 2016), and such chemicals are unlikely to be crucial in mating behavior.

To summarize, we performed a comprehensive analysis of the expression of the olfactory-related genes during the *D. punctatus* mating process and annotated olfactory-related proteins relatively comprehensively. Considerable numbers of OBP, CSP, and OR genes with expression patterns correlated with mating behaviors were identified, including a group of *Dendrolimus* male specific ORs, which are candidate pheromone receptors. Furthermore, we identified several OBP and OR genes that were upregulated after mating in females, which may be those responsible for host location via plant volatiles. These results represent the first step toward comprehensive understanding of the olfactory mechanisms of *Dendrolimus* species, and the foundation for population control of this pest insect.

## Author contributions

SZ designed and carried out the laboratory experiments, sequence assemblies, and drafted the manuscript. HW and XK collected the insects in the field. FL performed part of the data analysis. ZZ designed the experiments and modified the manuscript.

### Conflict of interest statement

The authors declare that the research was conducted in the absence of any commercial or financial relationships that could be construed as a potential conflict of interest.

## Supplementary material

Figure S1 Quantitative real time PCR (qPCR) validation of transcriptome data. Expression levels were determined based on fragments per kb per million reads (FPKM), qPCR data are presented as means ± *SD*. **(A)** OR genes; **(B)** OBP genes.

Figure S2 Distribution of unigene sizes in the transcriptome assembly of *D. punctatus*.

Figure S3 Annotation distribution of transcriptome contigs from *D. punctatus* antennae transcriptomes. **(A)** GO analysis; **(B)** KOG classification.

Figure S4 GO enrichment of differentially expressed unigenes (DEGs) from *D. punctatus* with different mating status.

Figure S5 Expression pattern of *D. punctatus* odorant binding proteins (OBPs) in insects with different mating status. **(A)** OBPs expressed at higher levels in male than female antennae. **(B)** OBPs expressed at higher levels in female than male antennae. **(C)** OBPs exhibiting relatively high expression levels in insects with different mating status, but without sexual bias.

Figure S6 Expression pattern of *D. punctatus* chemosensory proteins (CSPs) in insects with different mating status. **(A)** CSPs expressed at higher levels in male than female antennae. **(B)** CSPs expressed at higher levels in female than male antennae. **(C)** CSPs exhibiting relatively high expression level in insects with different mating status, but without sexual bias.

Figure S7 Expression pattern of *D. punctatus* odorant receptors (ORs) in insects with different mating status. **(A)** ORs expressed at higher levels in male than female antennae. **(B)** ORs expressed at higher levels in female than male antennae. **(C)** ORs exhibiting relatively high expression levels in insects with different mating status, but without sexual bias.

Figure S8 Expression patterns of candidate *D. punctatus* gustatory receptors (GR) in insects with different mating status.

Figure S9 Expression patterns of candidate *D. punctatus* ionotropic receptors (IR) in insects with different mating status.

Table S1 The NCBI Accession number of updated and newly Identified sensory genes.

Table S2 Primers used for Real-time PCR of selected genes.
