# Supplementary figures and images for: Dynamic Changes in Chemosensory Gene Expression during the Dendrolimus punctatus Mating Process
Source: Front Physiol. 2018 Jan 10;8:1127. doi: 10.3389/fphys.2017.01127 (PMC5767605; doi:10.3389/fphys.2017.01127)

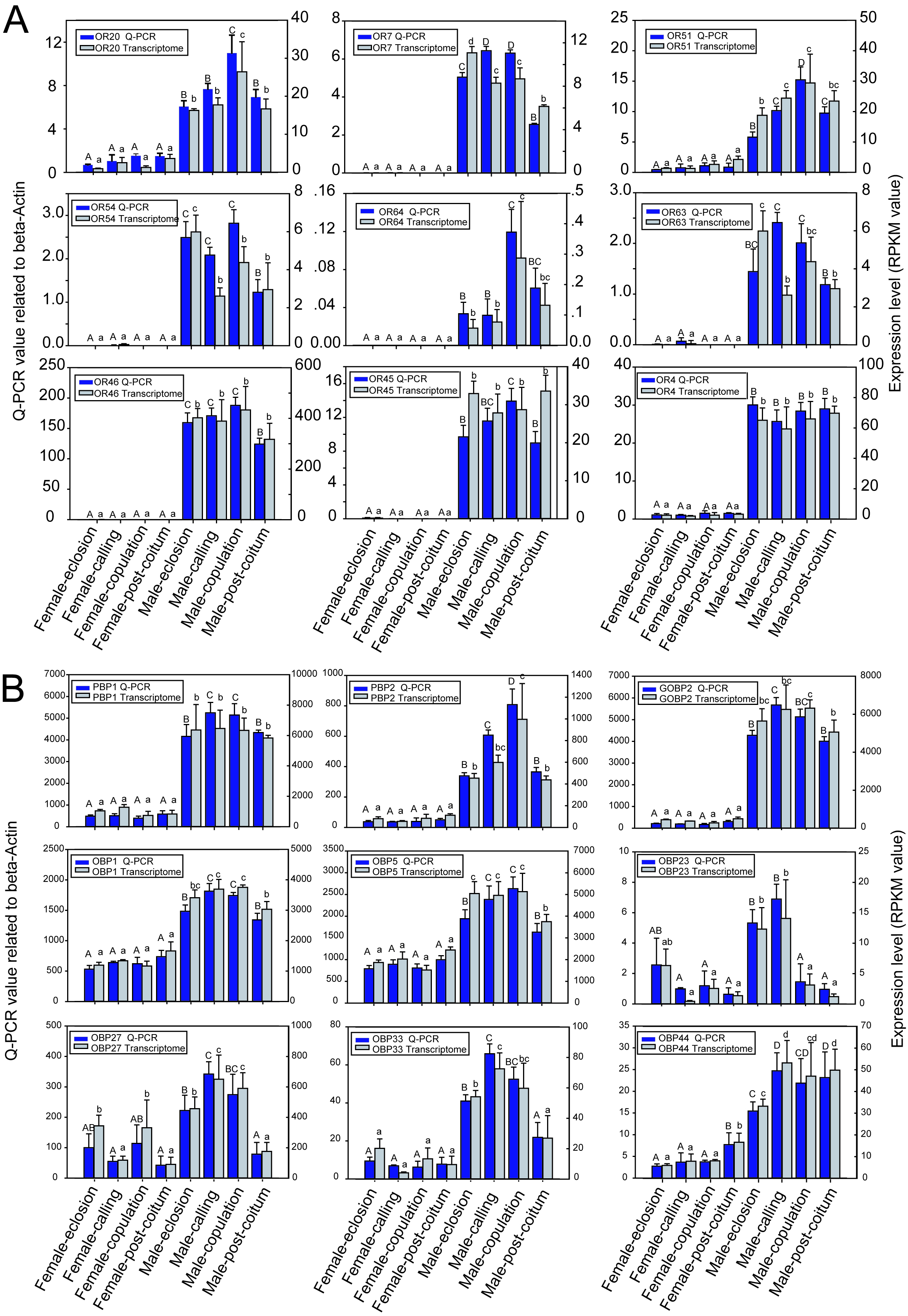

Supplement: Figure S1 — Quantitative real time PCR (qPCR) validation of transcriptome data. Expression levels were determined based on fragments per kb per million reads (FPKM), qPCR data are presented as means ± SD. (A) OR genes; (B) OBP genes. [file Image1.JPEG]

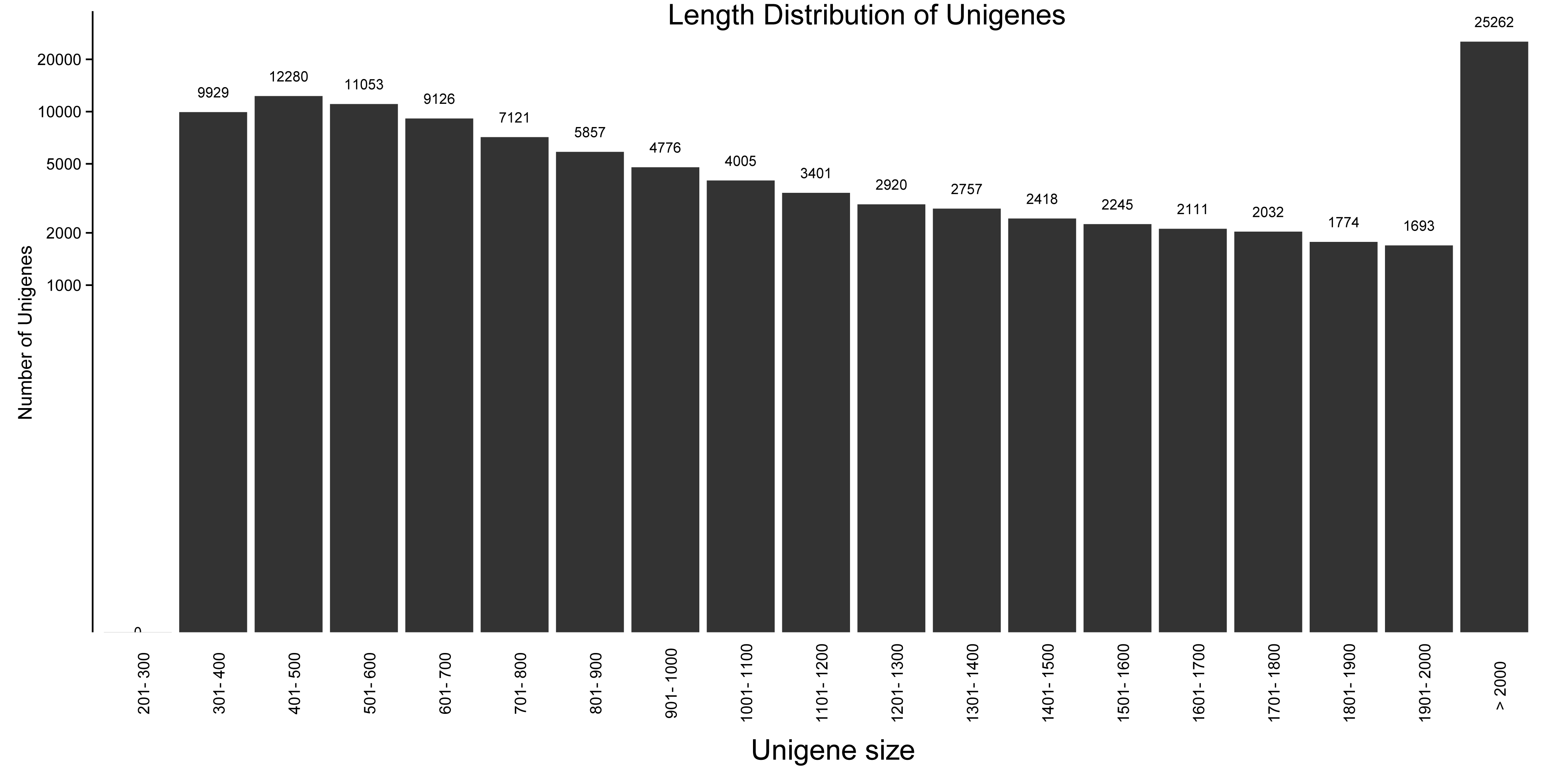

Supplement: Figure S2 — Distribution of unigene sizes in the transcriptome assembly of D. punctatus. [file Image2.JPEG]

A

## gene ontology classification

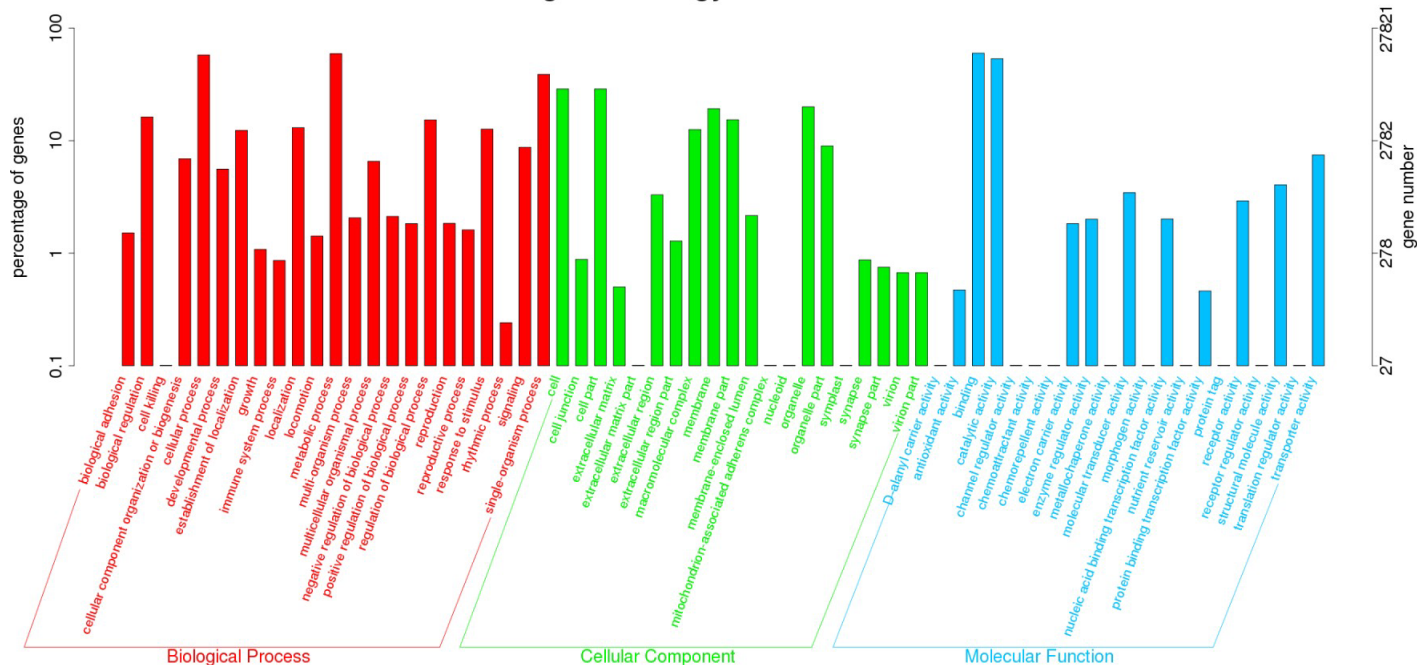

B

## KOG Function Classification

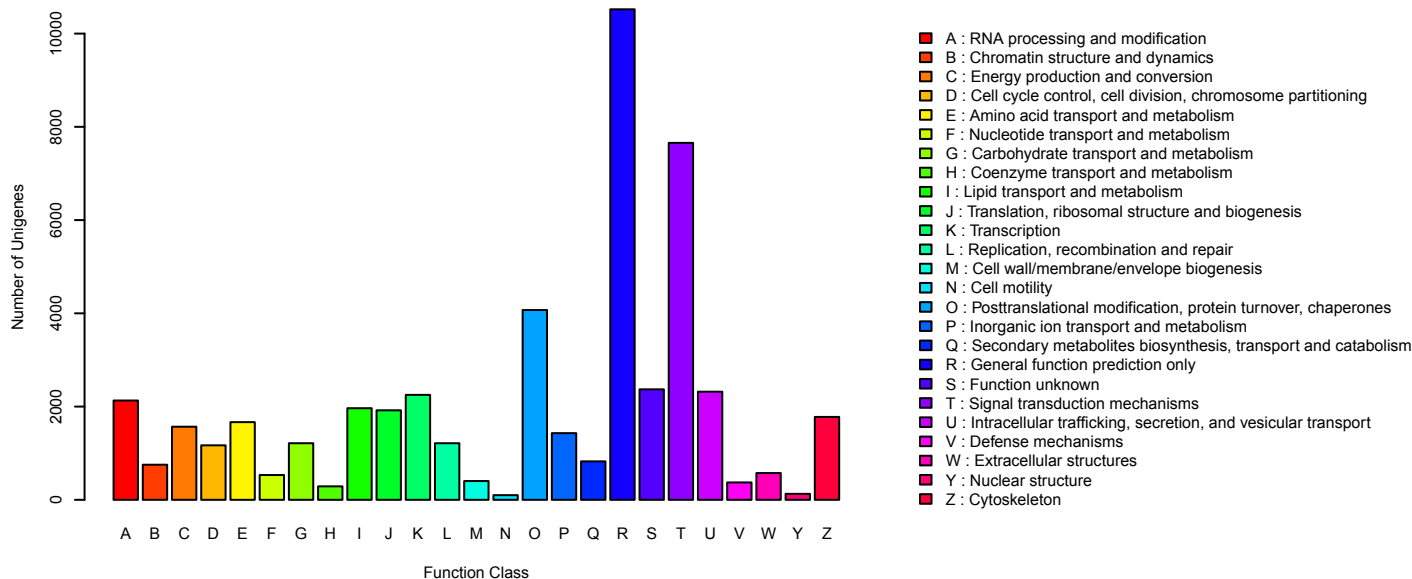

Supplement: Figure S3 — Annotation distribution of transcriptome contigs from D. punctatus antennae transcriptomes. (A) GO analysis; (B) KOG classification. [file Image3.PDF]

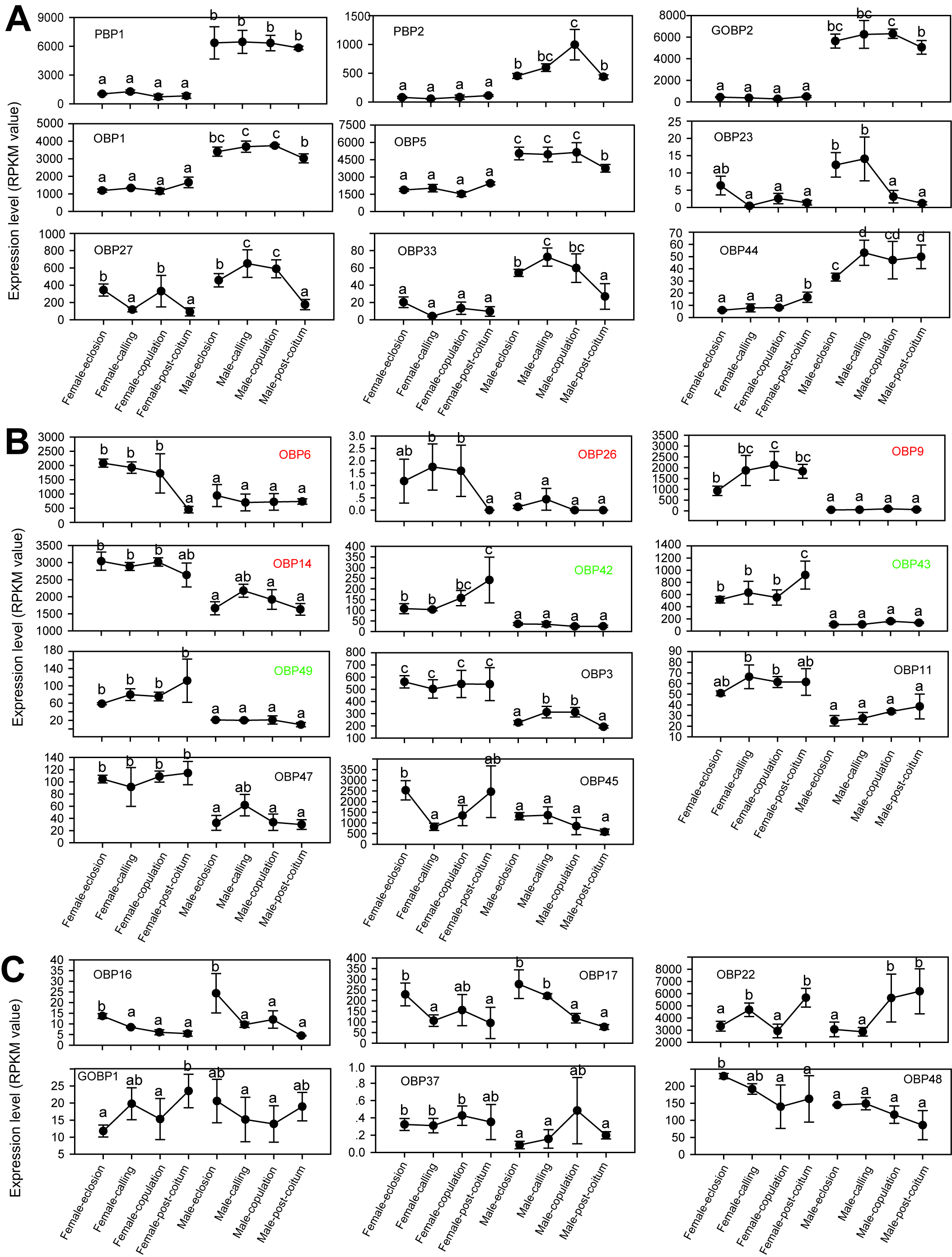

Supplement: Figure S5 — Expression pattern of D. punctatus odorant binding proteins (OBPs) in insects with different mating status. (A) OBPs expressed at higher levels in male than female antennae. (B) OBPs expressed at higher levels in female than male antennae. (C) OBPs exhibiting relatively high expression levels in insects with different mating status, but without sexual bias. [file Image5.JPEG]

**A**

Expression level (RPKM value)

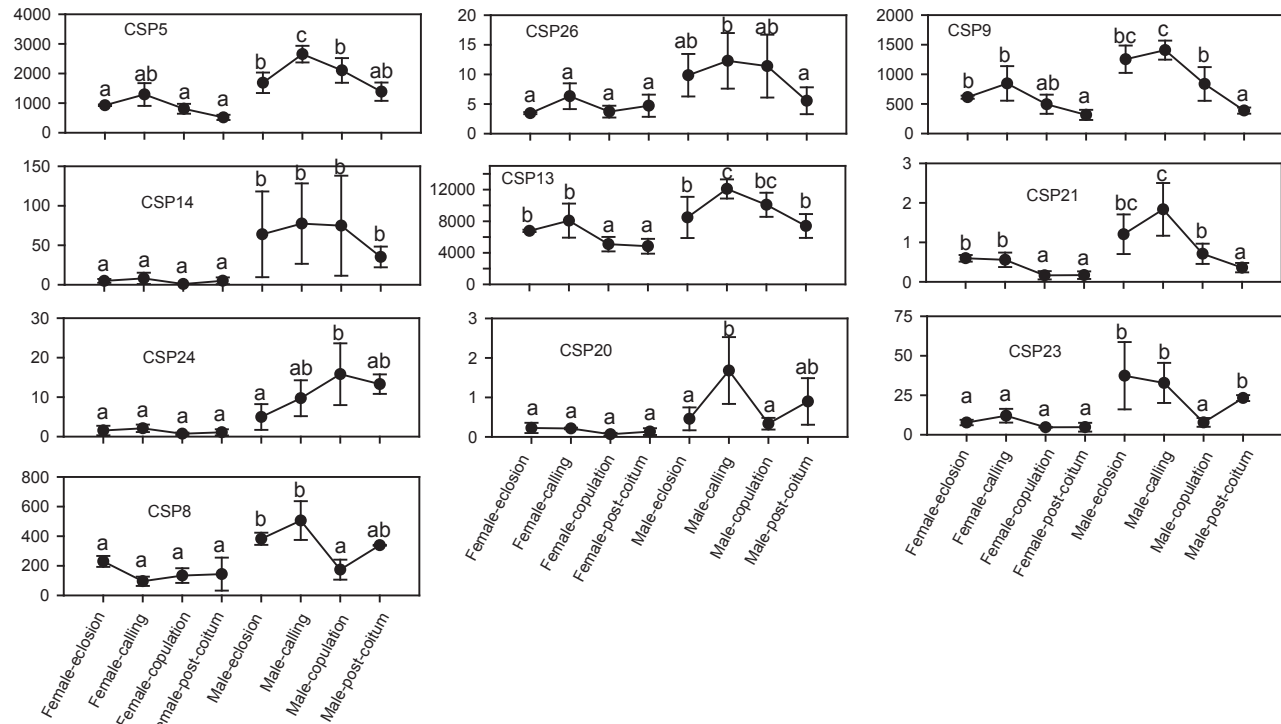**B**

Expression level (RPKM value)

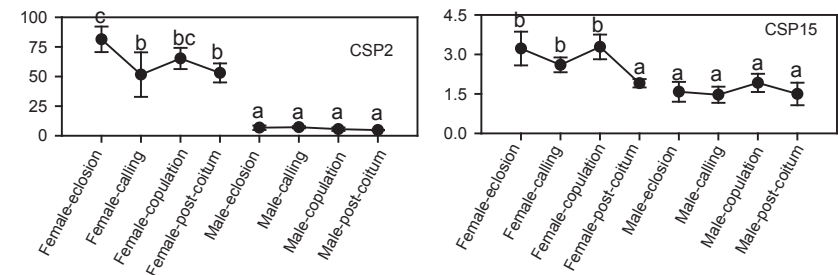**C**

Expression level (RPKM value)

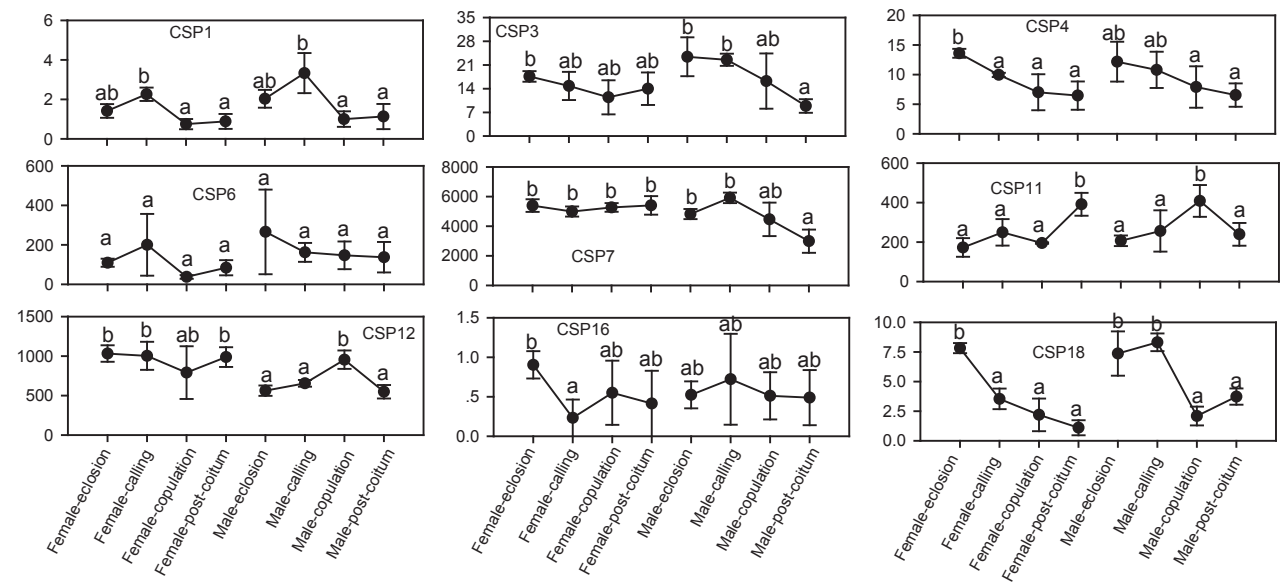

Supplement: Figure S6 — Expression pattern of D. punctatus chemosensory proteins (CSPs) in insects with different mating status. (A) CSPs expressed at higher levels in male than female antennae. (B) CSPs expressed at higher levels in female than male antennae. (C) CSPs exhibiting relatively high expression level in insects with different mating status, but without sexual bias. [file Image6.PDF]

A

Expression level (RPKM value)

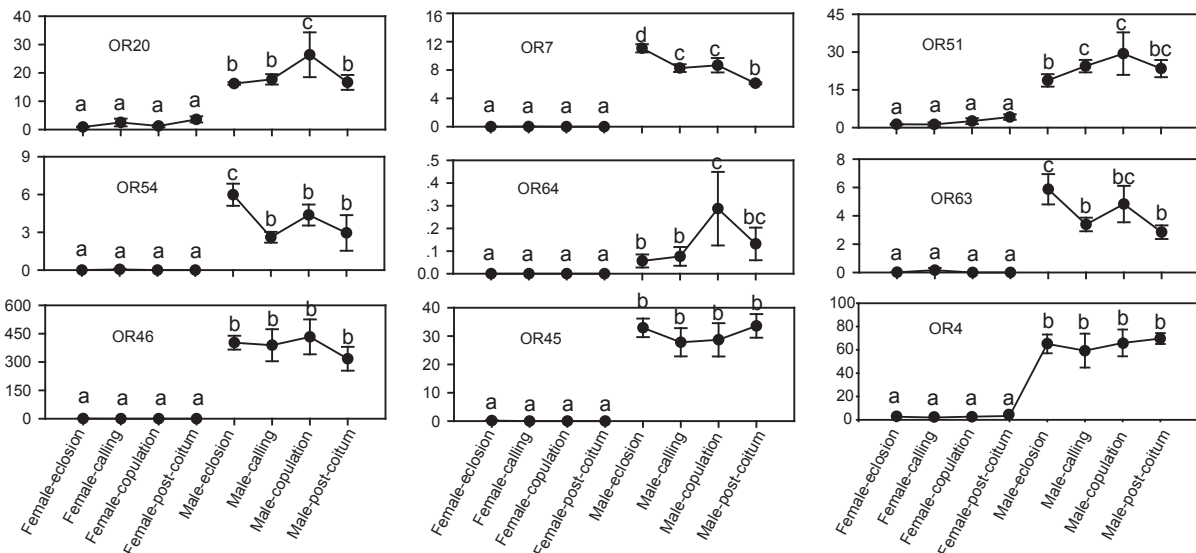

B

Expression level (RPKM value)

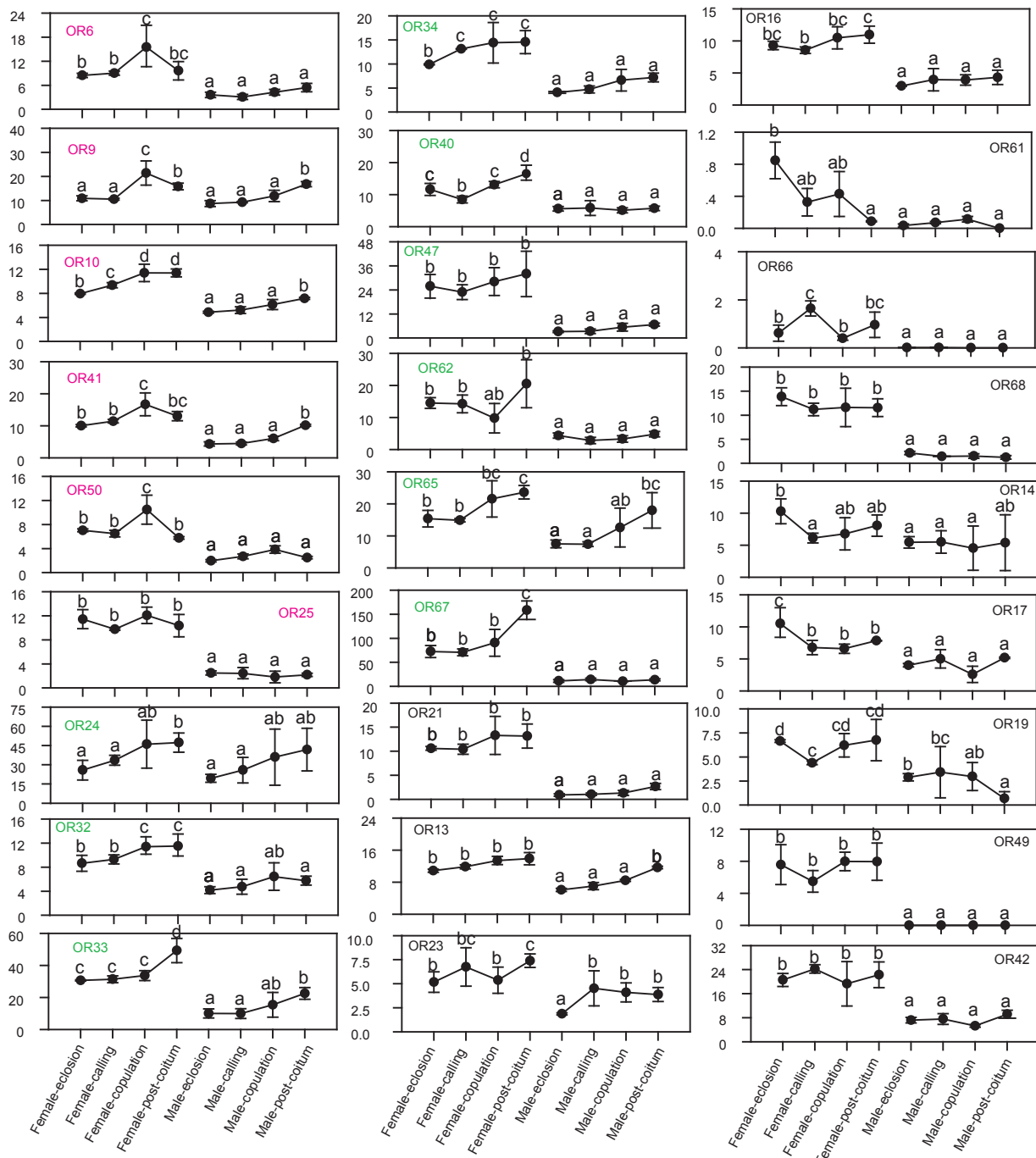

C

Expression level (RPKM value)

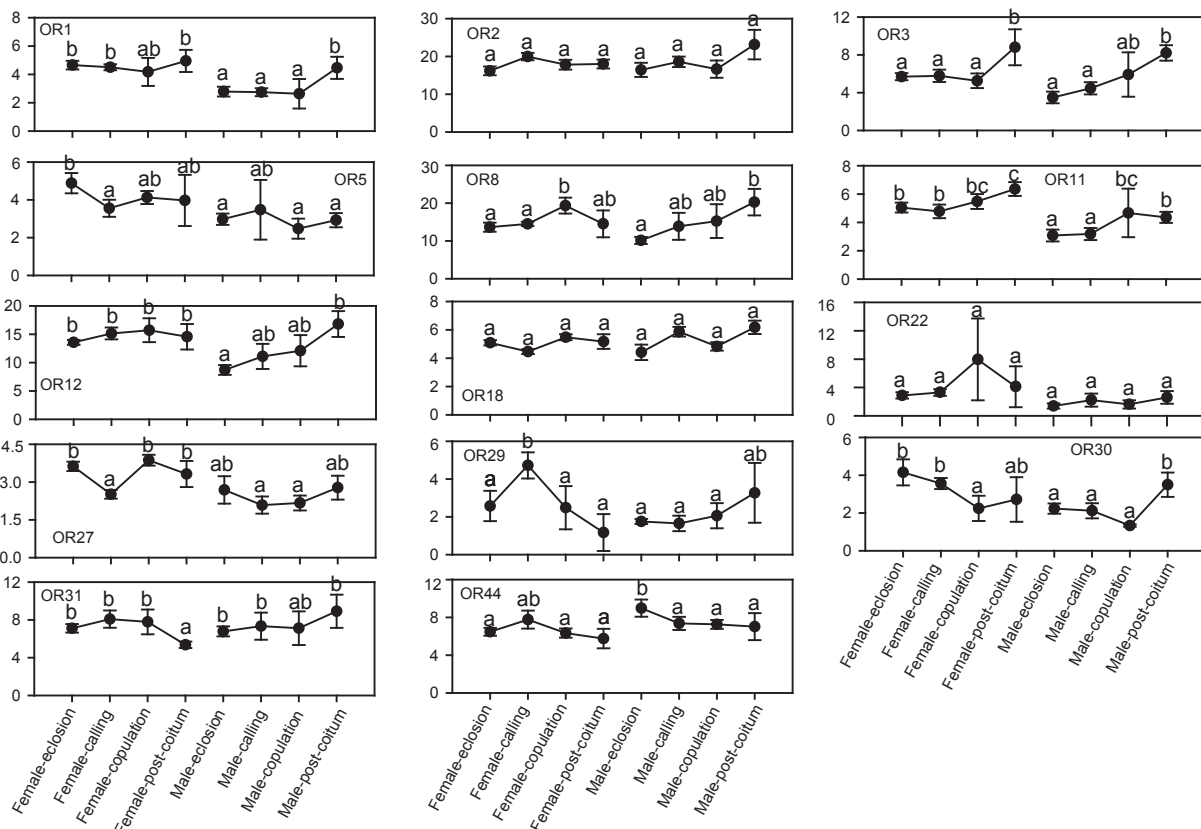

Supplement: Figure S7 — Expression pattern of D. punctatus odorant receptors (ORs) in insects with different mating status. (A) ORs expressed at higher levels in male than female antennae. (B) ORs expressed at higher levels in female than male antennae. (C) ORs exhibiting relatively high expression levels in insects with different mating status, but without sexual bias. [file Image7.PDF]

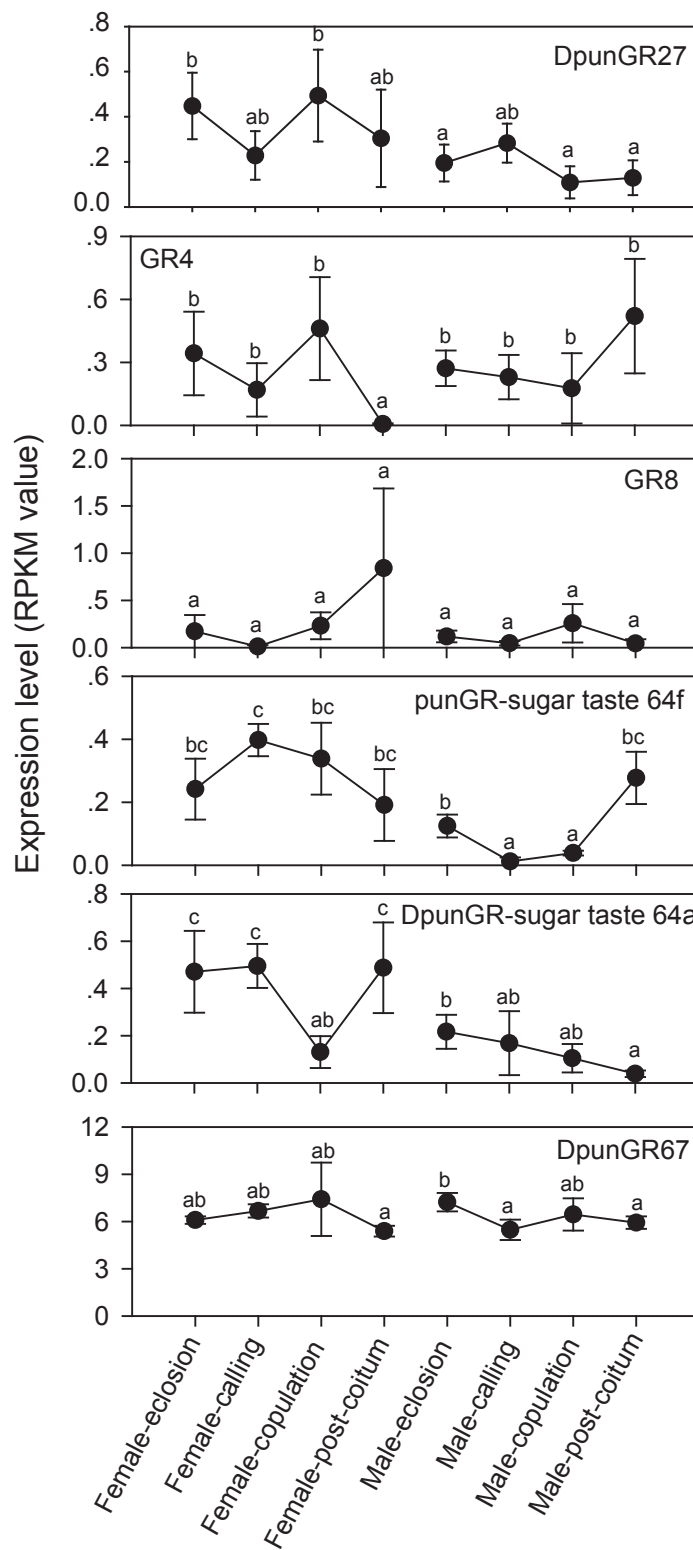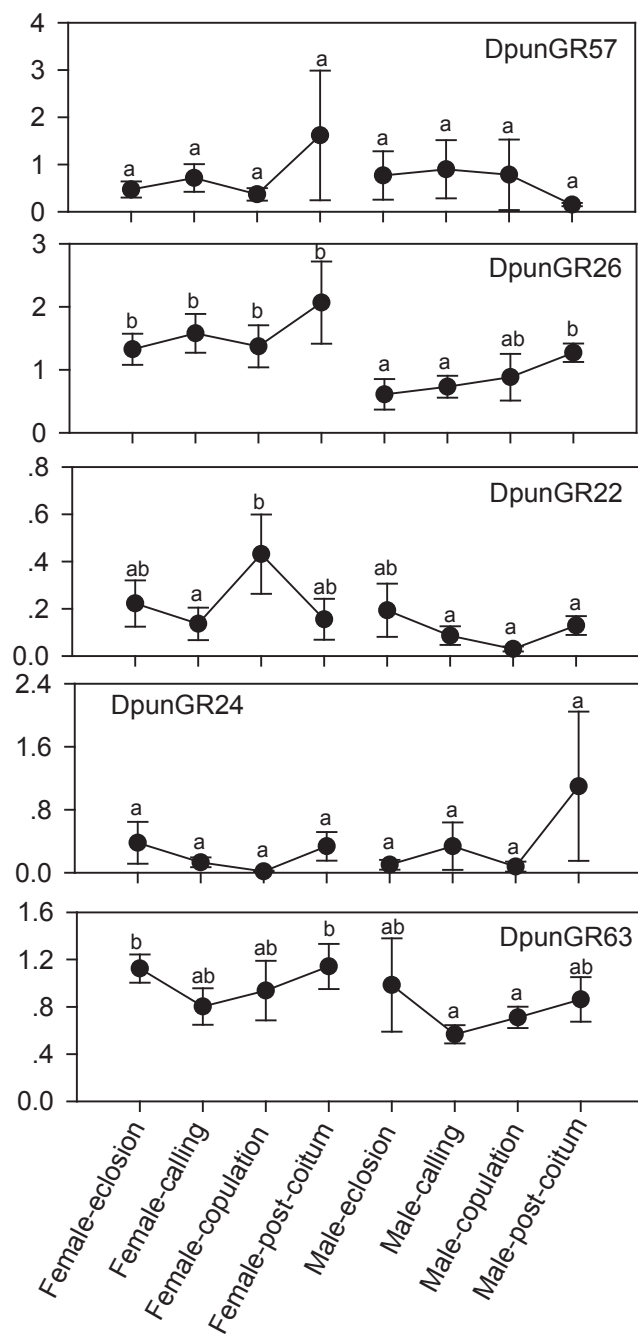

Supplement: Figure S8 — Expression patterns of candidate D. punctatus gustatory receptors (GR) in insects with different mating status. [file Image8.PDF]

Expression level (RPKM value)

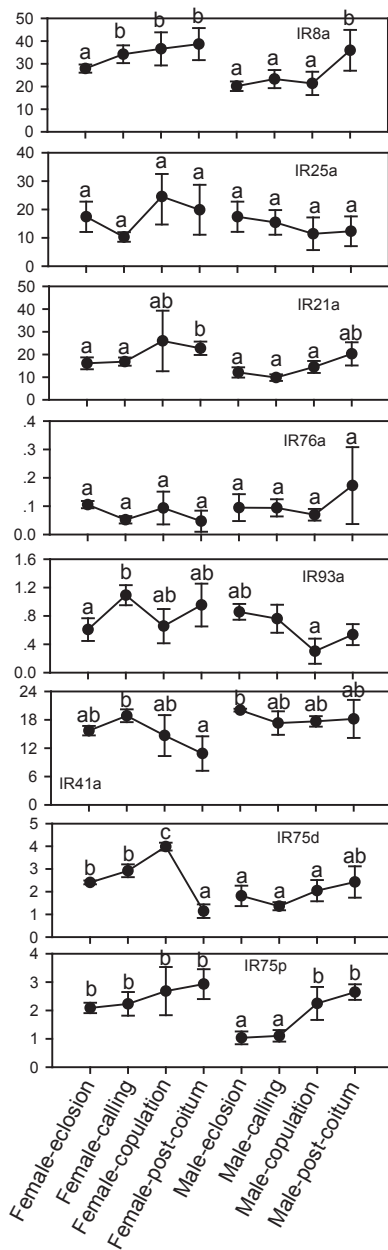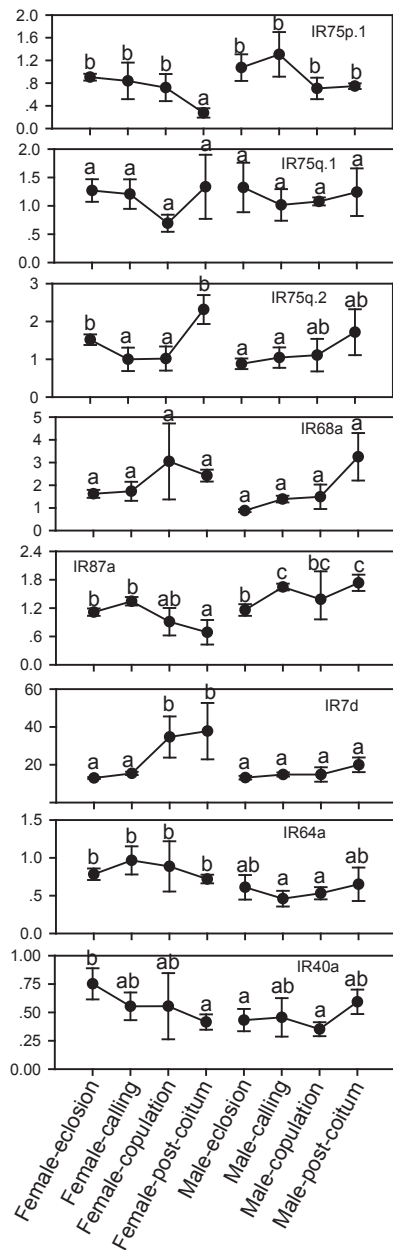

Supplement: Figure S9 — Expression patterns of candidate D. punctatus ionotropic receptors (IR) in insects with different mating status. [file Image9.PDF]
