# Supplementary material for: Dynamic Changes in Chemosensory Gene Expression during the Dendrolimus punctatus Mating Process
Source: Front Physiol. 2018 Jan 10;8:1127. doi: 10.3389/fphys.2017.01127 (PMC5767605; doi:10.3389/fphys.2017.01127)
Supplement: Figure S4 — GO enrichment of differentially expressed unigenes (DEGs) from D. punctatus with different mating status. [file Image4.PDF]

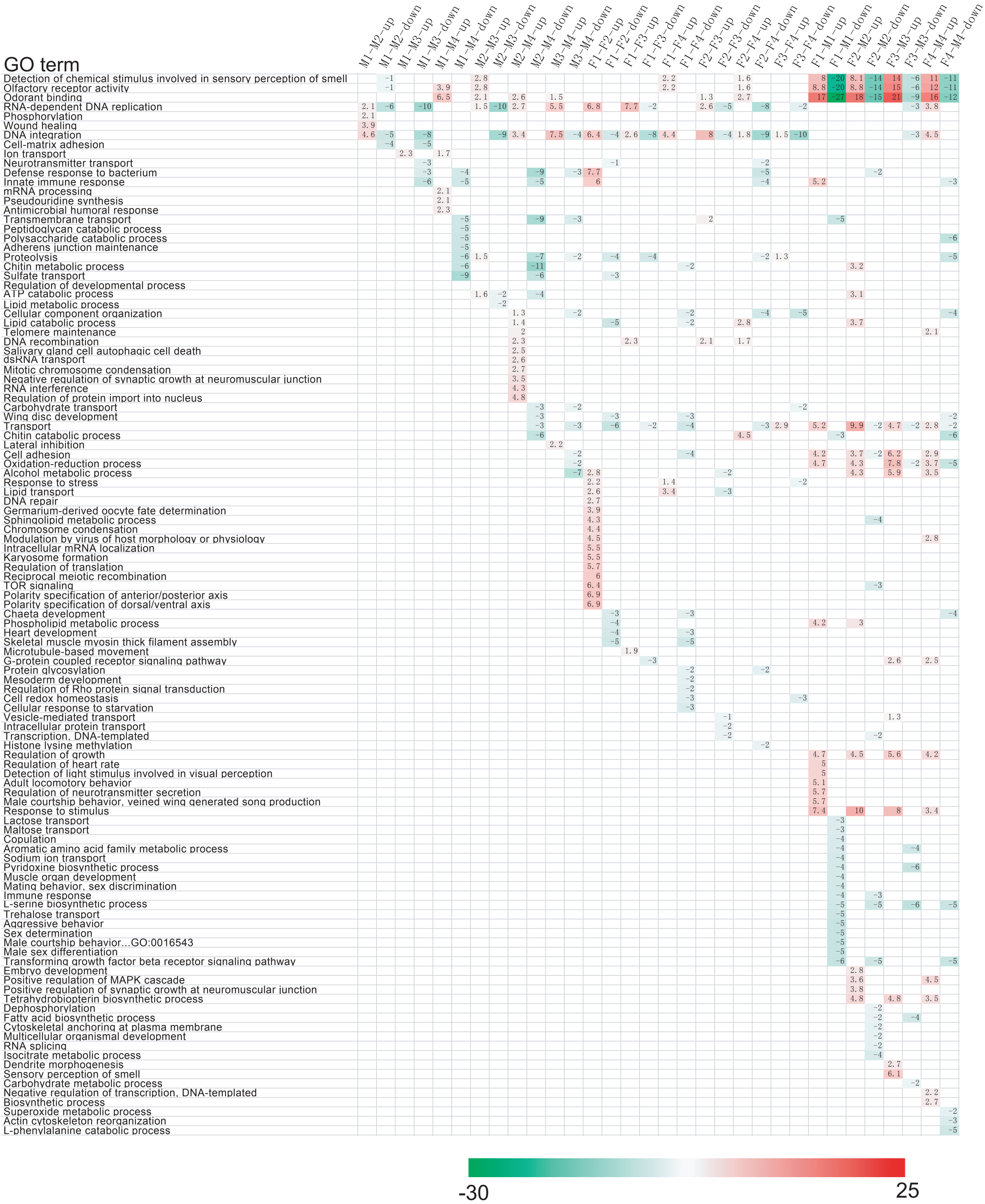
GO term
M1-M2-up
M1-M2-down
M1-M3-up
M1-M3-down
M1-M4-up
M1-M4-down
M2-M3-up
M2-M3-down
M2-M4-up
M2-M4-down
M3-M4-up
M3-M4-down
F1-F2-up
F1-F2-down
F1-F3-up
F1-F3-down
F1-F4-up
F1-F4-down
F2-F3-up
F2-F3-down
F2-F4-up
F2-F4-down
F3-F4-up
F3-F4-down
F1-M1-up
F1-M1-down
F2-M2-up
F2-M2-down
F3-M3-up
F3-M3-down
F4-M4-up
F4-M4-down
Detection of chemical stimulus involved in sensory perception of smell
Olfactory receptor activity
Odorant binding
RNA-dependent DNA replication
Phosphorylation
Wound healing
DNA integration
Cell-matrix adhesion
Ion transport
Neurotransmitter transport
Defense response to bacterium
Innate immune response
mRNA processing
Pseudouridine synthesis
Antimicrobial humoral response
Transmembrane transport
Peptidoglycan catabolic process
Polysaccharide catabolic process
Adherens junction maintenance
Proteolysis
Chitin metabolic process
Sulfate transport
Regulation of developmental process
ATP catabolic process
Lipid metabolic process
Cellular component organization
Lipid catabolic process
Telomere maintenance
DNA recombination
Salivary gland cell autophagic cell death
dsRNA transport
Mitotic chromosome condensation
Negative regulation of synaptic growth at neuromuscular junction
RNA interference
Regulation of protein import into nucleus
Carbohydrate transport
Wing disc development
Transport
Chitin catabolic process
Lateral inhibition
Cell adhesion
Oxidation-reduction process
Alcohol metabolic process
Response to stress
Lipid transport
DNA repair
Germarium-derived oocyte fate determination
Sphingolipid metabolic process
Chromosome condensation
Modulation by virus of host morphology or physiology
Intracellular mRNA localization
Karyosome formation
Regulation of translation
Reciprocal meiotic recombination
TOR signaling
Polarity specification of anterior/posterior axis
Polarity specification of dorsal/ventral axis
Chaeta development
Phospholipid metabolic process
Heart development
Skeletal muscle myosin thick filament assembly
Microtubule-based movement
G-protein coupled receptor signaling pathway
Protein glycosylation
Mesoderm development
Regulation of Rho protein signal transduction
Cell redox homeostasis
Cellular response to starvation
Vesicle-mediated transport
Intracellular protein transport
Transcription, DNA-templated
Histone lysine methylation
Regulation of growth
Regulation of heart rate
Detection of light stimulus involved in visual perception
Adult locomotory behavior
Regulation of neurotransmitter secretion
Male courtship behavior, veined wing generated song production
Response to stimulus
Lactose transport
Maltose transport
Copulation
Aromatic amino acid family metabolic process
Sodium ion transport
Pyridoxine biosynthetic process
Muscle organ development
Mating behavior, sex discrimination
Immune response
L-serine biosynthetic process
Trehalose transport
Aggressive behavior
Sex determination
Male courtship behavior...GO:0016543
Male sex differentiation
Transforming growth factor beta receptor signaling pathway
Embryo development
Positive regulation of MAPK cascade
Positive regulation of synaptic growth at neuromuscular junction
Tetrahydrobiopterin biosynthetic process
Dephosphorylation
Fatty acid biosynthetic process
Cytoskeletal anchoring at plasma membrane
Multicellular organismal development
RNA splicing
Isocitrate metabolic process
Dendrite morphogenesis
Sensory perception of smell
Carbohydrate metabolic process
Negative regulation of transcription, DNA-templated
Biosynthetic process
Superoxide metabolic process
Actin cytoskeleton reorganization
L-phenylalanine catabolic process
-30
25
