# Supplementary material for: Dynamic Changes in Chemosensory Gene Expression during the Dendrolimus punctatus Mating Process
Source: Front Physiol. 2018 Jan 10;8:1127. doi: 10.3389/fphys.2017.01127 (PMC5767605; doi:10.3389/fphys.2017.01127)
Supplement: Table S2 — Primers used for Real-time PCR of selected genes. [file Table2.docx]

**Table S2 Primers used for Real-time PCR of selected genes.**

| **Primer Name** | **Sequence (5'—3')** |
| --- | --- |
| DpuActinQ-5‘ | GCGATCTTACCGACTACCTCA |
| DpuActinQ-3‘ | TCTGGGCAACGGAACCT |
| DpuOR20Q-5‘ | AGTGACATCGTGGAAG |
| DpuOR20Q-3‘ | GTAACTGGACACCGTAC |
| DpuOR7Q-5‘ | ACTTGATAAGTATTGGGTAC |
| DpuOR7Q-3‘ | TAGCGTTGTTTGATGTT |
| DpuOR51Q-5‘ | TTCCGAGTGCGATAT |
| DpuOR51Q-3‘ | CGTTTACCAGGTTCAT |
| DpuOR54Q-5‘ | ACGGTGATTTACTTATGG |
| DpuOR54Q-3‘ | TGGCTGTAGGCTGGA |
| DpuOR64Q-5‘ | CGGAAATTAGGCTGTA |
| DpuOR64Q-3‘ | CATCCACGTTGTCGT |
| DpuOR63Q-5‘ | TTAGCCAATCCCATCC |
| DpuOR63Q-3‘ | ACGCCATTTCTGAAGG |
| DpuOR46Q-5‘ | ATTGTGGTTGGGAGTG |
| DpuOR46Q-3‘ | GCGTATCAAGGCAGA |
| DpuOR45Q-5‘ | ATTCTTGGGCATACTTC |
| DpuOR45Q-3‘ | CAATCACTGCGGACA |
| DpuOR4Q-5‘ | AAACAATCGGGAAAC |
| DpuOR4Q-3‘ | ACTCGTAACAGCGTAAT |
| DpuPBP1Q-5‘ | CGTGTCTGCGGACTTCT |
| DpuPBP1Q-3‘ | TTGTGCGTGTTATCCTTGT |
| DpuPBP2Q-5‘ | AAGAGTATGACAGCCAGTT |
| DpuPBP2Q-3‘ | ATAATCCTCACGCCAAT |
| DpuGOBP2Q-5‘ | ACAAGTTCTCCCTCCTCCA |
| DpuGOBP2Q-3‘ | GGGCGATTCCCTCTTTC |
| DpuOBP1Q-5‘ | ACGGATTTGTAGCAGA |
| DpuOBP1Q-3‘ | AACTTCCTCACCCTTT |
| DpuOBP5Q-5‘ | AAACAAACGTGGACTTATC |
| DpuOBP5Q-3‘ | ATTACACCGTCAGACATCA |
| DpuOBP23Q-5‘ | CGAACGGCTTCTTCC |
| DpuOBP23Q-3‘ | TGGCGAGTTATCTTGGT |
| DpuOBP27Q-5‘ | GGCTTCGCTTCCTCT |
| DpuOBP27Q-3‘ | TGTATTCATCGCTATCCTC |
| DpuOBP33Q-5‘ | TCACCCTATCAAGAACA |
| DpuOBP33Q-3‘ | CATCGACTGGCGTAT |
| DpuOBP44Q-5‘ | GGGGTCACAGAAGAGG |
| DpuOBP44Q-3‘ | CATGTGGCTCGCTCT |
